# Propensity for performing interventions in pre-hospital trauma management – a comparison between physicians and non-physicians

**DOI:** 10.1186/1752-2897-8-3

**Published:** 2014-02-07

**Authors:** Mathias C Blom, Ludwig Aspelin, Kjell Ivarsson

**Affiliations:** 1 IKVL, Medicine, Lund University, IKVL/Avd för medicin, Hs 32, EA-blocket, plan 2, Universitetssjukhuset, Lund SE 221 85, Sweden; 2 Ortopediska kliniken, Falu lasarett, Landstinget Dalarna, Box 712, Falun SE 791 29, Sweden; 3 Head of division: “Specialiserad närsjukvård Skånevård SUND”, Medicon Village, Tunavägen 3, Lund SE 22363, Sweden

**Keywords:** Trauma, Medical services, Emergency, Emergency care, Prehospital

## Abstract

**Background:**

In 2005, the Advanced Life Support (ALS) teams delivering pre-hospital care in RegionSkane in southern Sweden received additional support by physicians, who were part of “Pre-hospital acute teams” (PHAT). The study objective is to compare the incidence of pre-hospital medical interventions for trauma-patients cared for by conventional ALS teams and patients who received additional support by PHAT.

**Methods:**

Trauma patients with Injury Severity Score (ISS) >9 were identified retrospectively in the national quality registry KVITTRA at three hospitals in RegionSkane, for the time period October 2005 to December 2008. Interventions include e.g. tracheal intubation, administration of i.v. fluids, neck immobilization and spine board usage. Confounding effects from trauma severity, trauma mechanism, vital parameters, age and sex were addressed in multivariate models.

**Results:**

Data from 202 cases was included. 9 pre-hospital interventions were assessed. The incidence of endotracheal intubation and immobilisation of extremities was higher among patients in the PHAT-group compared to the ALS-only group (16.3% vs. 6.9%, p = 0.034) and (12.8% vs. 4.3%, p = 0.027) respectively. PHATs presence remained a significant predictor of these interventions also after taking confounding factors into account (OR 5.5, CL 1.5-19.7) and (OR 3.2 CI 1.0-9.8).

PHAT was involved in a greater proportion of cases with <50.0% of survival (19.8% vs. 12.1%, p = 0.134). The average ISS was higher among cases receiving PHAT support in strata ISS 16-24 and ISS > 24 than cases in corresponding strata cared for by ALS teams alone (ISS 20.0 vs. 17.0, p = 0.048 and ISS 34.0 vs. 29.0, p = 0.019).

**Conclusions:**

The incidence of endotracheal intubation and immobilization of extremities was greater among patients supported by PHAT, compared to patients cared for by ALS teams alone. This finding has to be interpreted in the light of a selection-bias where PHAT support was directed to more severely injured patients.

## Introduction

Scientific evaluation of physicians caring for trauma patients in the pre-hospital setting is not fully conclusive [1]. There is some support for a beneficial effect of their presence [2], despite of the contradictory results of a large multi-centre study [3]. A randomized controlled trial addressing the subject suggests that physicians adhere to pre-hospital intervention protocols better and succeed in a larger number of interventions than nurses do. The same study showed lower mortality rates among patients receiving pre-hospital care from physicians [4]. Beneficial effects of physician presence have also been shown for patients suffering blunt head trauma [5]. Other studies have failed to show a difference [6]. Several studies show that physicians tend to be directed to trauma of higher severity [7-9].

A recent study comparing physicians and ambulance nurses highlighted several physician-specific competencies [10]. Others have shown that trauma-patients cared for by physicians are more prone to be treated and discharged at the trauma scene [11]. Still others argument that it is more important to identify need and to perform an intervention correctly, than which profession possesses a certain competence [12]. Evaluating physicians in pre-hospital care by addressing outcomes has been questioned, as intra-hospital factors might confound results [10].

Advanced Life Support (ALS) is the pre-hospital system used in the country council RegionSkane in southern Sweden. The ALS-teams consist of a nurse and an emergency medicine technician. Starting in 2005, the ALS teams received support by “Pre-hospital acute teams” (PHAT), staffed by a physician and a nurse. One PHAT-team was stationed at each of the general hospitals in Helsingborg, Kristianstad, Lund and Malmoe. PHAT was introduced at slightly different times at the different hospitals (Helsingborg general hospital: October 2005, Kristianstad general hospital: March 2006, Skane University hospital, Lund: January 2005 and Malmoe general hospital: October 2006). In December 2008, PHAT was abolished simultaneously in all hospitals due to a perceived lack of benefits. From October 2005 to December 2008 PHAT was the only means by which ALS teams could receive pre-hospital support by a physician.

The study objective is to evaluate whether the incidence of pre-hospital interventions differs between trauma-patients cared for by conventional ALS teams compared to patients who received additional support by PHAT. Bearing work performed by others in mind, the authors believe that the incidence of interventions would possibly be higher among patients who received support by PHAT. The present study will add important information about pre-hospital physician support under Swedish conditions, which (according to the knowledge of the authors) has not yet been scientifically evaluated.

## Methods

### Study design

The study was conducted as a register study, utilizing data retrieved from the quality registry KVITTRA (Kvalitet i Traumavården). Trauma patients were identified in the local KVITTRA registries of Helsingborg general hospital, Kristianstad general hospital and Skane University hospital, Lund. Malmoe general hospital was not connected to KVITTRA and was therefore not included.

### Outcomes

The main outcome of the study was differences in incidence of pre-hospital medical interventions between trauma-patients cared for by conventional ALS teams and trauma-patients that received additional support from PHAT. Differences in 30-day mortality were the secondary outcome.

### Setting

RegionSkane is a country council in southern Sweden, populated by about 1 100 000 people, few of which live further than 50 km from the closest hospital. There is no full Level-I trauma centre in RegionSkane. Skane University Hospital, Lund is the only hospital with access to neurosurgery and thoracic surgery around the clock, while both Skane University hospital, Lund and Malmoe general hospital have access to vascular surgery. Malmoe general hospital is the only hospital having access to hand-surgery around the clock. Other hospitals in the region have to refer patients to these levels of care.

Physician members of PHAT were senior residents or specialists in anesthesiology, emergency medicine, internal medicine or surgery. Exceptions were one primary care physician and one otolaryngologist. All had undergone ATLS training, as well as specific training provided by RegionSkane. PHAT was dispatched either upon request by the ALS teams, by the alarm-center staff (in accordance with criteria stated in the list of PHAT dispatching criteria) or on PHATs own accord. The latter was possible as PHAT had access to radio-communication between the alarm-center and the ALS teams. PHAT could also give advice over telephone. Empirical knowledge suggests that PHAT was consulted over telephone in the majority of trauma cases of ISS > 15.

List of PHAT dispatching criteria

1. Cardiac arrest or suspected cardiac arrest

2. Suspected very traumatized patient

3. Unconscious patient with obvious signs of affected breathing and circulation

4. Lack of ambulances in the vicinity

5. Request from a conventional ALS-team

6. Need for care directorship or at an incident with more than five patients suffering severe trauma

7. PHAT personnel find dispatching necessary, e.g. for educational purposes

KVITTRA is a national quality registry for trauma-care, encompassing trauma-patients who die within 30 days of the trauma, undergo surgery within 24 hours, receive care in the ICU or are transported to a regional hospital. Data is registered in KVITTRA prospectively. For each hospital in RegionSkane connected to KVITTRA, one person (nurse or secretary) is responsible for the registrations. Only cases with ISS > 9 are exported to the national registry. In addition to pre-hospital interventions, KVITTRA also includes vital parameters, primary- and secondary injury mechanisms (ICD-9/10), AIS, ISS, TRISS and some information about intra-hospital interventions (e.g. time to x-ray investigation). Registered vital parameters are measured in the pre-hospital setting. KVITTRA does not contain data on the anatomic distribution of injuries. In RegionSkane, many cases with ISS < 10 are registered in KVITTRA as they are referred to Malmoe for hand-surgery.

Due to low priority of registrations in KVITTRA at Skane University Hospital, Lund, only 8 months (Jan-Aug 2008) of the study period were covered in the Lund registry. All pre-hospital interventions registered in KVITTRA could be performed by PHAT physicians as well as by the ALS teams, except from blood transfusion, which was therefore not evaluated.

### Inclusion criteria

Patients with Injury Severity Score (ISS) >9, who were registered in KVITTRA at the the general hospitals of Helsingborg and Kristianstad, and Skane University hospital, Lund during the period PHAT was in use and who arrived to the hospital by ambulance were eligible for study. Cases subject to incomplete data on 30-day mortality, physician attendance or primary trauma-mechanism were excluded.

### Determining sample size

To be able to detect a 20% effect size, each study arm has to contain 70 cases according to the formula below:

$$n=\frac{2{\left(Z_{a}+Z_{1-b}\right)}^{2_{\propto }2}}{\Delta^{2}}$$

*n* = sample size

*Z*_*a*_ = 1.96 for two sided effect, p = 0.05 level

*Z*_1-*b*_ = 0.8416 for 80% power

∝ = 0.40 (estimated sample SD)

Δ = effect size

All patients that met the inclusion criteria were eligible for inclusion, in order not to introduce additional bias.

### Data collection

Data on physician attendance, pre-hospital interventions and baseline characteristics in the form of 30-day mortality, in-patient length of stay (IPLOS), time spent on the trauma-scene, trauma-mechanism (according to ICD9/10), age, sex, vital parameters (respiratory rate, Glasgow Coma Scale and systolic blood-pressure), ISS and TRISS were collected from KVITTRA and merged using Microsoft Office Excel® Mac 2011 (Microsoft Corporation, USA). Statistical analysis was performed using IBM® SPSS® Statistics 19. Prevented likely deaths were quantified as surviving cases with probability of survival <50.0% and unlikely deaths were defined as cases who died despite of having >50.0% probability of survival (based on the TRISS methodology).

### Differences in baseline characteristics and pre-hospital interventions

Continuous variables were assessed for normality using Shapiro-Wilks test. Differences in number of patients with low probability for survival, prevented likely deaths and unlikely deaths as well as ISS, age, time spent on the trauma scene and IPLOS were assessed using the Mann-Whitney U test. Comparison of the incidence of pre-hospital interventions was performed using the Chi2 test and Fisher exact test where appropriate. Due to established dispatching-criteria, a selection bias was expected. In order to compensate for part of this bias, binary logistic regression models addressing confounding from age, sex, trauma severity (ISS), trauma mechanism, vital parameters and 30-day mortality were constructed.

### Variables

ISS and age were categorized according to intervals used in the Major Trauma Outcome Study [13], i.e. ISS 10-15, ISS 16-24, ISS > 24 and age dichotomized at 55 years. Vital parameters were categorized according to intervals used in the Revised Trauma Score (RTS) methodology. However, some groups were merged due to their small size. Reference intervals in the model were: GCS score 13-15, Respiratory rate 10-29 breaths/min, ISS 10-15. To enter the final model, a variable had to be more strongly associated with the outcome than p = 0.10. Associations with p < 0.05 were considered statistically significant. Trauma-mechanisms according to ICD-9/10 were manually classified as “blunt trauma” or “penetrating trauma” by one of the authors (MB).

Ethical approval for the study was granted KI from the ethical approval committee of Lund.

## Results

### Included patients

A total of 621 cases were registered in KVITTRA at the three hospitals during the study period (Helsingborg, n = 247, Kristianstad, n = 115, Lund, n = 259). Of the 621 screened cases, 362 had an ISS < 10 and were not eligible for inclusion.

Of the 259 remaining cases, 1 did not arrive by ambulance, 21 lacked data on physician attendance, 29 lacked data on 30-day mortality, and 5 lacked data on trauma-mechanism. One unreasonable case was identified (registered as dead within 30 days but registered IPLOS was 172 days). These 57 cases were excluded and hence 202 patients entered the final analyses. From the previously stated formula for sample size calculation, this would be enough to detect an effect size of 19%.

### Baseline characteristics

Out of the 202 cases, 86 (43%) patients received support from PHAT and 116 (57%) were cared for by the ALS teams alone. 22 (25.6%) cases receiving support by PHAT died within 30 days, compared to 18 (15.5%) cases in the other group (p = 0.076).

Patients supported by PHAT had a significantly higher median ISS than patients cared for by ALS teams alone among cases with ISS 16-24 (20.0 vs. 17.0, p = 0.048) and ISS > 24 (34.0 vs. 29.0, p = 0.019). Median IPLOS for patients receiving support from PHAT and having ISS > 24 was less than for patients attended to by the ALS teams alone (0.5 days vs. 13.0 days, p = 0.27). No statistically significant differences in patient age or time spent on the trauma scene were seen. 19.8% of patients cared for by PHAT had a probability of survival of <50.0%, compared to 12.1% for patients cared for by the ALS teams alone (p = 0.134). 3.5% of patients in the PHAT-group survived despite of having <50.0% probability of survival, vs. 1.7% in the ALS-only group (p = 0.43). 9.3% of patients in the PHAT-group died despite of having >50.0% probability of survival, compared to 5.2% in the ALS-only group (p = 0.254). For further details, please see Table 1.

**Table 1:** Baseline characteristics

	**ISS 10-15**			**ISS 16-24**			**ISS > 24**			**Total**		
	**Non-physician (n = 38)**	**Physician (n = 31)**	**p**	**Non-physician (n = 47)**	**Physician (n = 25)**	**p**	**Non-physician (n = 31)**	**Physician (n = 30)**	**p**	**Non-physician (n = 116)**	**Physician (n = 86)**	**p**
ISS (IQR)	13.0 (3.0)	12.0 (3.0)	0.29	17.0 (3.0)	20.0 (5.5)	0.048	29.0 (8.0)	34.0 (16.5)	0.019	17 (12)	20 (16)	0.34
Age, years (IQR)	44.0 (36.8)	47.0 (47.0)	0.23	48.0 (37.0)	48.0 (27.5)	0.67	41.0 (33.0)	37.0 (33.5)	0.78	44 (35.8)	44.5 (35.5)	0.49
Male	26	25	0.25	28	16	0.71	22	23	0.61	76	64	0.18
Female	12	6	0.25	19	9	0.71	9	7	0.61	40	22	0.18
Time on trauma scene, hh:mm (IQR) (N missing)	00:19 (00:11) (13)	00:18 (00:14) (0)	0.61	00:15 (00:10) (18)	00:14 (00:11) (0)	0.40	00:15 (00:11) (4)	00:12 (00:14) (2)	0.39	00:16 (00:10) (35)	00:15 (00:14) (2)	0.82
IPLOS, days (IQR)	5.5 (12.5)	9.0 (13.0)	0.058	4.0 (14.0)	9.0 (19.0)	0.083	13.0 (28.0)	0.5 (24.5)	0.27	5.5 (16)	7.5 (18.3)	0.24
Penetr. trauma	2	2	1.0	3	1	1.0	0	4	0.053	5	7	0.26
GCS (IQR)	15 (1)	15 (0)	0.32	14 (8)	15 (2.5)	0.032	13 (12)	6.5 (12)	0.302	14 (6.75)	15 (8)	0.34
Systolic b.p., mmHg (IQR)	130 (42.8)	139 (35)	0.75	120 (41)	130 (54.5)	0.16	100 (50)	105 (77.8)	0.53	120 (41)	130 (51.5)	0.18
Resp. rate, breaths/min (IQR)	20 (4)	20 (4)	0.73	16 (4)	20 (3.5)	0.001	20 (10)	16 (13.3)	0.98	20 (4)	20 (4.25)	0.17
30 day mort.	0 (0.0%)	1 (3.2%)	0.45	8 (17.0%)	4 (16.0%)	1.0	10 (32.3%)	17 (56.7%)	0.073	18 (15.5%)	22 (25.6%)	0.076
N.o. cases with <50 pct prob. of survival	0 (0%)	0 (0%)	1.0	4 (8.5%)	1 (4.0%)	0.48	10 (32.3%)	16 (53.3%)	0.099	14 (12.1%)	17 (19.8%)	0.134
Prevented likely deaths	0 (0%)	0 (0%)	1.0	1 (2.1%)	0 (0%)	0.47	1 (3.2%)	3 (10.0%)	0.29	2 (1.7%)	3 (3.5%)	0.43
Unlikely deaths	0 (0%)	1 (3.2%)	0.27	5 (10.6%)	3 (12.0%)	0.86	1 (3.2%)	4 (13.3%)	0.15	6 (5.2%)	8 (9.3%)	0.254

Median and inter-quartile range (IQR). Mann-Whitney U test used for comparisons. Missing values indicated where appropriate.

### Prehospital interventions

The incidence of endotracheal intubation was higher among patients supported by PHAT, compared to patients attended to by ALS teams alone (16.3% vs. 6.9%, p = 0.034). This effect remained significant for patients with ISS > 24 (40.0% vs. 16.1%, p = 0.038) upon subgroup analysis. The incidence of immobilization of extremities was also higher among patients attended to by PHAT (12.8% vs. 4.3%, p = 0.027). A trend to higher incidence of administration of crystalline- and colloid fluids was also seen 52.3% vs. 39.7% (p = 0.074) and 3.5% vs. 0.0% (p = 0.076) respectively. Results above are not adjusted for confounding. One patient, who was cared for by the ALS teams only, was subject to blood transfusion according to the registry. This was outside the therapeutic possibilities of the ALS teams and is attributed to registration error. For further details, please see Table 2.

**Table 2:** Incidence of pre-hospital interventions stratified according to trauma severity and in total

	**ISS 10-15**			**ISS 16-24**			**ISS > 24**			**Total**		
**Intervention**	**ALS only (n = 38)**	**PHAT (n = 31)**	**p**	**ALS only (n = 47)**	**PHAT (n = 25)**	**p**	**ALS only (n = 31)**	**PHAT (n = 30)**	**p**	**ALS only (n = 116)**	**PHAT (n = 86)**	**p**
Nasal airway	2	1	1.0	1	0	1.0	0	2	0.24	3	3	0.70
Oral airway	0	1	0.45	3	1	1.0	2	3	0.67	5	5	0.75
Endotrach. intubation	0	0	-	3	2	1.0	5	12	0.038	8	14	0.034
Neck-immob.	32	27	0.74	32	20	0.28	25	21	0.33	89	68	0.69
Spine board	30	23	0.64	26	17	0.30	24	18	0.14	80	58	0.82
Immob. of extremity	2	5	0.23	1	2	0.28	2	4	0.43	5	11	0.027
Crystalline fluids	13	15	0.23	14	11	0.23	19	19	0.87	46	45	0.074
Colloid fluids	0	0	-	0	1	0.35	0	2	0.24	0	3	0.076
Hypertonic solution	0	0	-	0	0	-	0	1	0.49	0	1	0.24

Chi2 and Fisher’s exact test used for comparing incidence.

After taking confounding factors into account, the probability of endotracheal intubation remained higher in the PHAT-group compared to the ALS-only group (OR 5.5, Cl 1.5-19.7). The same holds true for immobilization of extremity (OR 3.2 CI 1.0-9.8). No other significant differences between the compared groups were seen in the multivariate analysis. For further details, please see Table 3.

**Table 3:** Adjusted probability for pre-hospital interventions

**Intervention**	**Strength of association of PHAT presence to intervention (p)**	**OR for experiencing intervention if cared for by PHAT (Cl)**	**Fit of regression model (Nagelkerke R**^**2**^**)**
Nasal airway	≥ 0.10	-	0.092
Oral airway	≥ 0.10	-	0.225
Endotracheal intubation	0.01	5.5 (1.5-19.7)	0.521
Neck-immobilisation	≥ 0.10	-	0.464
Spine board	≥ 0.10	-	0.405
Immobilisation of extremity	0.041	3.2 (1.0-9.8)	0.129
Crystalline fluids	≥ 0.10	-	0.115
Colloid fluids	≥ 0.10	-	0.576
Hypertonic solution	≥ 0.10	-	0.704

Probability is expressed as odds ratio (OR), normalised for confounding from age, sex, trauma severity, trauma mechanism, vital parameters and 30-day mortality. OR shown for associations p ≤ 0.10.

## Discussion

The incidence of endotracheal intubation and immobilisation of extremities was higher among patients in the PHAT-group compared to the ALS-only group: 16.3% vs. 6.9% (p = 0.034) and 12.8% vs. 4.3% (p = 0.027) respectively. PHATs presence remained a significant predictor of these interventions also after taking confounding factors into account (OR 5.5, CL 1.5-19.7 and OR 3.2 CI 1.0-9.8, respectively). A trend to higher incidence of treatment with crystalline fluids (52.3% vs. 39.7%, p = 0.074) and colloid fluids (3.5% vs. 0.0%, p = 0.076) was seen in the PHAT-group. No differences in fluid therapy were seen in the multivariate analyses. The main results gain some support from other studies [4,11].

Limitations include that telephone consultations were not adjusted for, but might have influenced the results. Furthermore, the lack of complete registrations in KVITTRA of Skane University Hospital, Lund during part of the study period imposes questions on data quality and also reduces the power of the study.

The presence of a selection-bias is indicated by a higher median ISS among cases of ISS 16-24 (20.0 vs. 17.0, p = 0.048) and ISS > 24 (34.0 vs. 29.0 p = 0.019) in the PHAT-group than corresponding strata within the ALS-only group. The bias most likely results from PHAT being directed to more severe cases due to its specific dispatching criteria (Table 1). The bias gains further support by the presence of a greater fraction of patients with low probability of survival (TRISS <50%) in the PHAT-group (3.5% vs. 1.7%). This is most evident among patients of ISS > 24 (10.0% vs. 3.2%, p = 0.29). Even though it affects the results, the selection bias was expected and gains support in the literature [7-9]. The shorter IPLOS for cases with ISS > 24 in the PHAT-group compared to corresponding cases in the ALS-only group (0.5 vs. 13.0 days, p = 0.27) is most likely explained by the higher mortality in this group.

The authors believe that the results can be interpreted in either of two ways. The first is that PHAT and the associated more aggressive pre-hospital treatment is not beneficial to patients, as it is associated with higher 30-day mortality and more unlikely deaths. The other interpretation is that the higher mortality is caused by a selection bias, together with factors influencing 30-day mortality not taken into account by the TRISS methodology (e.g. co-morbidity). Even though an attempt to address the bias was made through stratifying the groups into ISS-intervals, this is most likely not enough to adequately discern subtle variation in risk within each stratum. Hence it still remains to be elucidated how much of the variation in pre-hospital treatment could be attributed to different competencies of the pre-hospital personnel.

## Conclusion

The incidence of endotracheal intubation and immobilization of extremities was greater among patients who received support by PHAT compared to patients cared for by ALS teams alone, in the present study. Due to the presence of a selection bias caused by PHAT being directed to trauma of greater severity, it remains to be elucidated what proportion of the results could be explained by different competencies of the pre-hospital personnel. It is desirable to couple differences in the provision of pre-hospital care to functional outcomes in future studies, which would require organ-specific data as well as data on intra-hospital factors and patient co-morbidity.

## Consent

Only anonymized data was analyzed and hence written consent was not obtained from individual patients. This procedure was supported by the decision from the regional ethical review board in Lund.

## Competing interests

The authors declare that they have no competing interests.

## Authors’ contributions

MB was the principal investigator and took part in the formulating the research question, in the statistical analyses, in searching the literature and in the writing of the final article. LA took part in formulating the research question, in searching the literature and in commenting on the manuscript. KI was the supervisor of the project. KI commented on repeated versions of the manuscript. KI provided support to the two other authors and answered clinical as well as methodological questions. KI also granted access to the data material used and was granted ethical approval for the study. All authors read and approved the final manuscript.

## References

[B1] Botker MT Bakke SA Christensen EF A systematic review of controlled studies: do physicians increase survival with prehospital treatment? Scand J Trauma Resusc Emerg Med 2009 17 12 PubMed PMID: 19265550. Pubmed Central PMCID: 2657098 10.1186/1757-7241-17-12 19265550 PMC2657098

[B2] Roudsari BS Nathens AB Cameron P Civil I Gruen RL Koepsell TD International comparison of prehospital trauma care systems Injury 2007 38 9 993 1000 PubMed PMID: 17640641. Epub 2007/07/21. eng 10.1016/j.injury.2007.03.028 17640641

[B3] Liberman M Mulder D Lavoie A Denis R Sampalis JS Multicenter Canadian study of prehospital trauma care Ann Surg 2003 237 2 153 160 PubMed PMID: 12560770. Pubmed Central PMCID: 1522139. Epub 2003/02/01. eng 12560770 10.1097/01.SLA.0000048374.46952.10 PMC1522139

[B4] Baxt WG Moody P The impact of a physician as part of the aeromedical prehospital team in patients with blunt trauma JAMA 1987 257 23 3246 3250 PubMed PMID: 3586248 10.1001/jama.1987.03390230082029 3586248

[B5] Lee A Garner A Fearnside M Harrison K Level of prehospital care and risk of mortality in patients with and without severe blunt head injury Injury 2003 34 11 815 819 PubMed PMID: 14580812. Epub 2003/10/29. eng 10.1016/S0020-1383(02)00395-9 14580812

[B6] Osterwalder JJ Mortality of blunt polytrauma: a comparison between emergency physicians and emergency medical technicians–prospective cohort study at a level I hospital in eastern Switzerland J Trauma 2003 55 2 355 361 PubMed PMID: 12913649. Epub 2003/08/13. eng 10.1097/01.TA.0000034231.94460.1F 12913649

[B7] Graf M Demartines N Harder F Scheidegger D Polytrauma: comparison of the hospital course after air- (with emergency physician) versus ground transport (without emergency physician) Helv Chir Acta 1993 59 4 649 653 PubMed PMID: 8473185. Epub 1993/03/01. Polytrauma: Vergleich des Spitalverlaufes nach Luft- (mit Notarzt) versus Bodentransport (ohne Notarzt). ger 8473185

[B8] Oppe S De Charro FT The effect of medical care by a helicopter trauma team on the probability of survival and the quality of life of hospitalised victims Accid Anal Prev 2001 33 1 129 138 PubMed PMID: 11189116. Epub 2001/02/24. eng 10.1016/S0001-4575(00)00023-3 11189116

[B9] Ringburg AN Spanjersberg WR Frankema SP Steyerberg EW Patka P Schipper IB Helicopter emergency medical services (HEMS): impact on on-scene times J Trauma 2007 63 2 258 262 PubMed PMID: 17693821. Epub 2007/08/19. eng 10.1097/01.ta.0000240449.23201.57 17693821

[B10] van Schuppen H Bierens J Understanding the prehospital physician controversy. Step 1: comparing competencies of ambulance nurses and prehospital physicians Eur J Emerg Med 2011 18 6 322 327 10.1097/MEJ.0b013e32834533f4 21460731

[B11] Roberts K Blethyn K Foreman M Bleetman A Influence of air ambulance doctors on on-scene times, clinical interventions, decision-making and independent paramedic practice Emerg Med J 2009 26 2 128 134 PubMed PMID: 19164630 10.1136/emj.2008.059899 19164630

[B12] Ummenhofer W Scheidegger D Role of the physician in prehospital management of trauma: European perspective Curr Opin Crit Care 2002 8 6 559 565 PubMed PMID: 12454542 10.1097/00075198-200212000-00013 12454542

[B13] Champion HR Copes WS Sacco WJ Lawnick MM Keast SL Bain LW Jr The major trauma outcome study: establishing national norms for trauma care J Trauma 1990 30 11 1356 1365 PubMed PMID: 2231804. Epub 1990/11/01. eng 10.1097/00005373-199011000-00008 2231804

